# Case report: pathological complete response in an IIIB non-small cell lung cancer patient after preoperative neoadjuvant nivolumab therapy combined with chemotherapy

**DOI:** 10.1097/MD.0000000000029336

**Published:** 2022-06-10

**Authors:** Luzhen Li, Ting Chen, Huiqin Lai, Ao Zhang, Xianhui Zhao, Jiaming Wu, Huisi Hong, Lexia Wu, Sihong Lin, Kexin Wang, Huatang Zhang, Cantu Fang

**Affiliations:** Zhongshan Affiliated Hospital, Guangzhou University of Chinese Medicine, Zhongshan, Guangdong Province, China.

**Keywords:** case report, immune checkpoint inhibitors, neoadjuvant therapy, nivolumab, non-small cell lung cancer

## Abstract

**Rationale:**

For locally advanced non-small cell lung cancer (NSCLC), the neoadjuvant therapy strategy of preoperative nivolumab combined with chemotherapy has great potential, especially for locally advanced NSCLC which are initially unresectable. They may be cured after neoadjuvant immunotherapy, and this may become a new direction of treatment. We hope that this representative medical record and literature review can provide some assistance for clinicians using immune checkpoint inhibitors to treat lung cancer.

**Patient concerns:**

A 50-year-old male patient was admitted to Zhongshan Hospital of Traditional Chinese Medicine on April 27, 2020 due to “coughing for more than one month.”. The patient had nothing of note in either his medical history or that of his family, and no history of smoking.

**Diagnosis:**

The diagnosis was cT4N2M0IIIB stage right lower lung NSCLC with right hilar and mediastinal lymph node metastasis. The stage was inoperable stage IIIB NSCLC, but the patient had a strong willingness for doing surgery.

**Interventions:**

The patient received 3 rounds of the neoadjuvant nivolumab therapy combined with TP (paclitaxel plus cisplatin) regimen, on 5-14-21, 06-07-21 and 07-07-21.

**Outcomes:**

The tumor's area shrunk. Then the patient underwent thoracoscopic radical resection of the cancer in the right upper lung and postoperative pathology achieved pathological complete response (pCR)

**Lessons:**

In this case, combined with the wishes of the patient and the latest research results, we confirmed pCR by radical surgery after 3 rounds of the neoadjuvant nivolumab therapy combined with chemotherapy. This may be a modality to cure more lung cancer patients in the future.

## 1 Introduction

Lung cancer is one of the most common malignant tumors in the world, and it is also the leading cause of death among malignant tumors.^[1]^ At present, conventional treatments have reached a bottleneck, while immunotherapy for lung cancer is booming. In recent years, immunotherapy has gradually become one of the 3 most important means of cancer treatment. Some programmed death-1 (PD-1)/programmed death receptor ligand-1 (PDL-1) inhibitors can activate the autoimmune system and inhibit tumor progression. As such, an increasing number of cancer patients have benefitted from immunotherapy.^[2,3]^ In recent years, great achievements have been made regarding immune checkpoint inhibitors in research on neoadjuvant immunotherapy. Among them, nivolumab's performance has been particularly eye-catching, bringing significant clinical benefits to some patients with early and middle stage tumors, and becoming a hot topic in the field of cancer treatment.^[4–9]^ We report a case of a patient with IIIB lung cancer. After 3 rounds of neoadjuvant nivolumab therapy combined with chemotherapy, we performed radical surgery to achieve pCR.

## 2 Case description

### 2.1 General conditions

**Figure 1:**
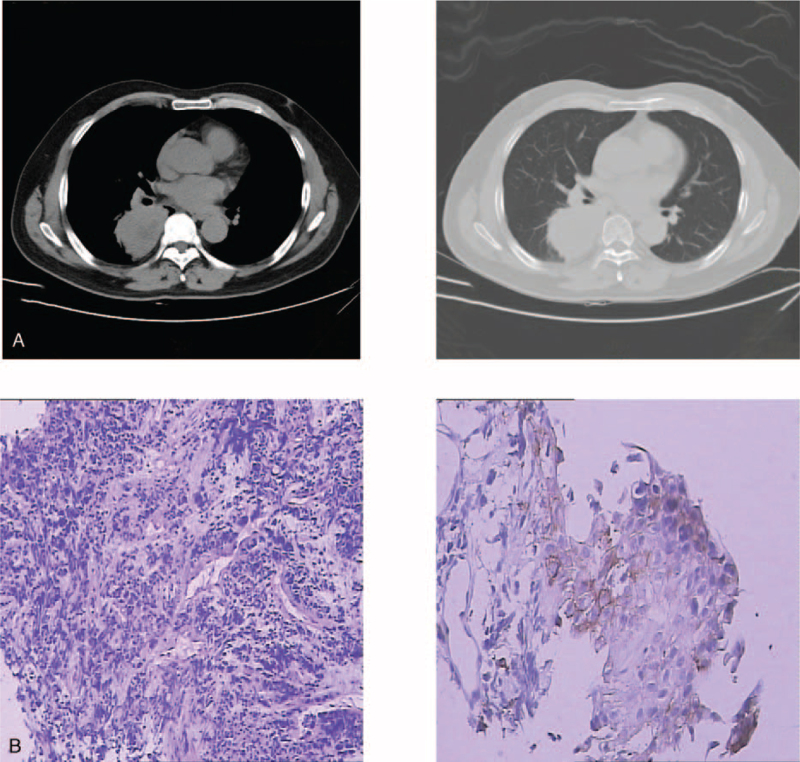
Data on initial diagnosis before neoadjuvant therapy 4-22-2021 (A): At first diagnosis, the CT showed that the lumpy soft tissue density shadow was in the dorsal segment of the lower lobe of the right lung. The area was about 69 mm × 56 mm, and there were many enlarged lymph nodes in the mediastinum, the length and diameter of which were about 19 mm. 05-01-2021 (B (IHC)): Immunohistochemistry: CK (+), Ki67 (60% +), p63 individual cells (+), TTF-1 (−), Syn (−), CD56 (−), CgA (−) (B (PD-L1)): PDL-1 TPS:2% (IHC is on the left; PD-L1 is on the right.).

A 50-year-old male patient was admitted to Zhongshan Hospital of Traditional Chinese Medicine on April 27, 2020 due to “coughing for more than one month.” He had with no special medical history or family medical history, and no smoking history. Physical examination: there was no palpable enlargement of the lymph nodes anywhere in his body. Cardiopulmonary physical examination showed nothing out of the ordinary. Imaging examination: (04–20–2021) enhanced CT conducted in Zhongshan Hospital of Traditional Chinese Medicine showed a lumpy soft tissue density shadow in the dorsal segment of the lower lobe of the right lung, with an area of about 69 mm × 56 mm. The edges were not smooth, the internal density was uneven, and the corresponding bronchus was occluded. Additionally, there were nodules in the middle lobe of the right lung, with a diameter of about 3 mm. The mediastinal shadow had no deviation, and there were many enlarged lymph nodes in the mediastinum, the length and diameter of which were about 19 mm. There was no pleural effusion, no pleural thickening and no destruction of bony thorax. We also found multiple cysts in the liver, the diameter of which were about 28 mm (Fig. 1A). Fiberoptic bronchoscopy biopsy showed: (right lower lung dorsal segment): non-small cell lung cancer, classified as poorly differentiated cancer; immunohistochemistry: CK (+), Ki67 (60% +), p63 individual cells (+), TTF-1 (-), Syn (-), CD56 (-), CgA (-), PDL-1 TPS: 2% (Fig. 1B); Genetic testing: EGFR (-), ALK (-), ROS1 (-).

### 2.2 Diagnosis and treatment process

The diagnosis was cT4N2M0IIIB stage of the right lower lung NSCLC with right hilar and mediastinal lymph node metastasis, inoperable stage IIIB non-small cell lung cancer. At present, the treatment mode with the highest level of evidence is sequential durvalumab immunoconsolidation therapy after simultaneous radiotherapy and chemotherapy.^[10–12]^ However, the patient had a strong willingness to proceed with surgery. After a multidisciplinary team consultation at Zhongshan Hospital of Traditional Chinese Medicine, we determined that the primary focus of the tumor was large and in close proximity to large blood vessels, such that radical surgery was not an option for the time being. According to the NADIM trial^[13]^ and the first positive PCR results obtained in the III phase clinical study of neoadjuvant immunotherapy in CheckMate816 reported orally at the 2021 American Association for Cancer Research annual meeting,^[14]^ this surgery is feasible with appropriate modes of chemotherapy and immunoadjuvant therapy. The expectation is that this treatment will simplify the surgery needed.^[13]^ After determining the treatment plan, we gave the patient 3 rounds of the neoadjuvant Nivolumab therapy combined with a TP regimen on 5–14–2021, 06–07–2021 and 07–07–2021. Practical usage: paclitaxel liposome 210 mg vd qd d1 + cisplatin injection 120 mg + nivolumab injection 360 mg vd qd d1. According to the CTCAE version 5.0,^[15]^ III-IV degree myelosuppression occurred in the patient after chemotherapy, which was relieved by symptomatic treatment.

## 3 Results

**Figure 2:**
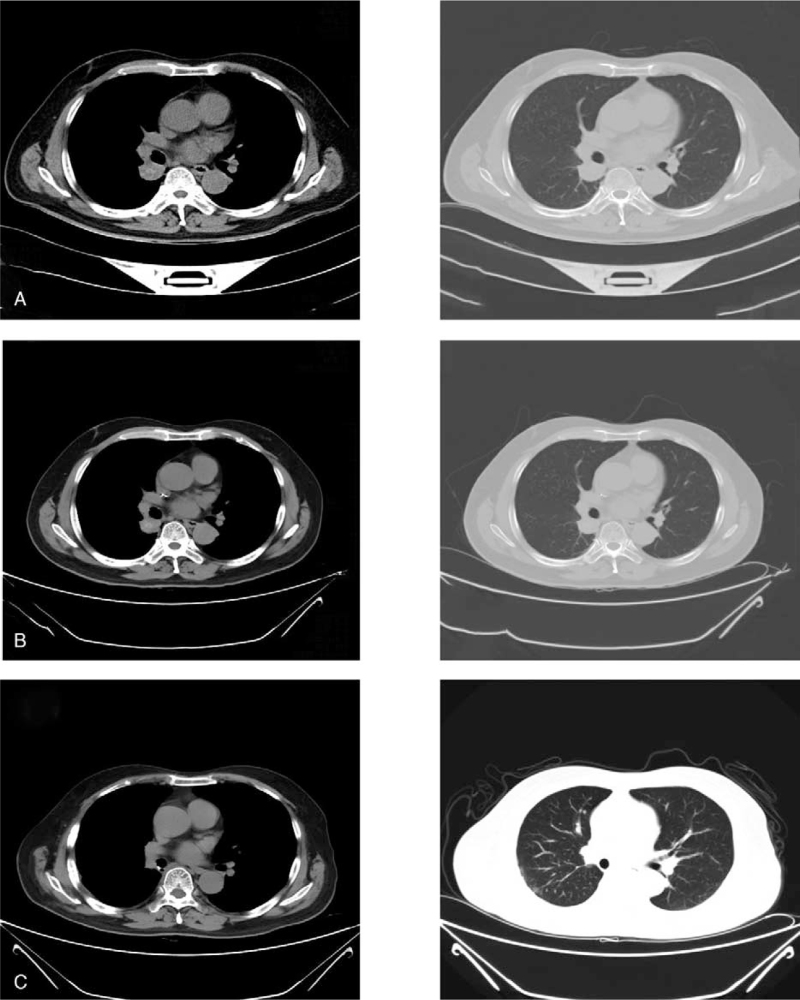
Data after neoadjuvant therapy 06-24-2021 (A): after 2 courses of neoadjuvant therapy, compared with the chest: the tumor in the dorsal segment of the lower lobe of the right lung was significantly smaller that of the CT imagery from 4-22-2021; the area was about 23 mm × 29 mm, the edge was smooth, and the surrounding exudate had been absorbed. The nodule in the middle lobe of the right lung was the same as before. The mediastinal and right hilar lymph nodes were smaller than before. 07-03-2021 (B): Before the third course of neoadjuvant therapy, the mass continued to shrink; the area was about 18 mm × 26 mm. 02-18-2022 (C): To date, we have observed no recurrence or obvious adverse reactions.

We conducted another chest-enhanced CT on 06–24–2021 after 2 courses of neoadjuvant therapy, and compared it with the chest CT imagery from 4-22-2021. The imagery showed that the tumor in the dorsal segment of the lower lobe of the right lung had shrunk to about 23 mm × 29 mm; the edge was smooth, and the surrounding exudate had been absorbed. The nodule in the middle lobe of the right lung was the same as before. Additionally, the mediastinal and right hilar lymph nodes were smaller than before. The condition of the rest of chest showed little change (Fig. 2A). After the third neoadjuvant therapy, the tumor shrank further (Fig. 2B). Then on August 4, 2021, the patient underwent thoracoscopic radical resection of the right upper lung tumor at the Sun Yat-Sen University Oncology Hospital. Postoperative pathology: we observed patchy necrosis, fibrous tissue hyperplasia with infiltration of lymphocytes, plasma cells, foam cells, multinucleated giant cell reaction, and cholesterol crystallization with calcification in the lung tissue. According to the RECIST version 1.1 response criteria, pathological complete response (pCR) had been achieved.^[16]^ We followed up on the patient every 3 months, for a total follow-up period of 8 months. The latest images showed no recurrence (Fig. 2C).

## 4 Discussion

Unlike chemotherapy and targeted drugs, the most important feature of immunotherapy is that its target is not the cancer cells themselves; rather, it returns the cancer patient's immune function to normal, and utilizes the host's immune response to inhibit and kill tumor cells. It also has high efficiency and low toxicity.^[17]^ However, the NSCLC staging is complex. Heated debate persists over the issue of determining the optimal treatment mode for maximizing the benefit of patients with stage III NSCLC. This case reveals the immense potential of immunotherapy with nivolumab in treating locally-advanced NSCLC patients. In the next section, combined with the results of the latest clinical studies, we review and analyze nivolumab neoadjuvant therapy for NSCLC. This offers us the opportunity to explore the neoadjuvant therapy mode of preoperative immunochemotherapy. Hopefully, this will enable more patients with early- and middle-stage-NSCLC to receive greater clinical benefits.

### 4.1 The application of nivolumab combined with chemotherapy in neoadjuvant therapy for NSCLC

The Checkmate159 study was the first clinical study to use immunotherapy in the field of neoadjuvant therapy for NSCLC.^[18]^ A total of 21 untreated, resectable early (Stage I, II or IIIA) NSCLC patients were enrolled in this clinical trial. We administered 2 doses of PD-1 inhibitor nivolumab (3 mg/kg) intravenously every 2 weeks before surgery, and surgery was scheduled for about 4 weeks after the first dose. The main endpoints of this study were safety and feasibility, with case remission, PD-L1 expression level, tumor mutation load and mutation-related new antigen-specific T cell response as secondary and exploratory endpoints. The results showed that of the 21 patients, 2 (10%) had partial remission, 18 (85%) had stable disease, and only 1 (5%) had progression. Long-term follow-up showed that the 20 patients who had received radical surgery had a disease-free recurrence rate of 73% after 18 months, and the overall survival rate reached as high as 95%.

The NADIM study is an open-label, multicenter, single-arm, 2-phase clinical study that includes histopathologically/cytopathologically confirmed, untreated and potentially resectable patients with stage IIIA NSCLC. The subjects in this study were resectable IIIA stage (N2, T4N0/N1), EGFR/ALK negative NSCLC patients. A total of 46 patients were enrolled in the group, and all patients received at least 1 cycle of neoadjuvant therapy. The preliminary data were published at the 19th World Lung Cancer Congress, and the data were updated at many major conferences such as ASCO and WCLC. According to the preliminary data, 83% of the patients with major pathologic response (MPR) achieved complete pathological remission (defined as no tumor cells being found), and 90% of the patients had decreased staging. The updated follow-up data were published in the journal *Lancet Oncology* on September 24, 2020. The data showed that all patients were removed by R0, and the 12-month progress free survival (PFS) rate was 95.7%. The PFS rate at 18 months was 87%, and the PFS rate at 24 months was 77.1%. In the revised intended treatment group (ITT) population, the overall survival (OS) rates of 12 months, 18 months and 24 months were 97.8%, 93.5%, and 89.9%, respectively.^[13]^ At the 2021 WCLC meeting, the researchers presented data from the NADIM trial with a median follow-up of 37.9 months. The updated data showed that in the ITT, the PFS rate for neoadjuvant therapy with nivolumab combined with chemotherapy for 36 months and 42 months was the same, both 69.6%. This suggested that there had been no disease progression over these 6 months. In the population treated according to the scheme (PP), the PFS rates of 36 months and 42 months were the same, both 81.8%. The OS rates were 91.0% and 87.3%, respectively, almost three times the previous survival figures.

CheckMate816 was the first 3-phase study to confirm the benefit of neoadjuvant therapy with immunotherapy plus chemotherapy for resectable NSCLC.^[14]^ Its primary endpoints were pCR and event-free survival. The secondary endpoints were OS, primary pathological remission (MPR, residual tumor cells ≤10%) and time to death or distant metastasis. Exploratory endpoints included outcomes such as surgical feasibility and perioperative related adverse events. The results showed that the pCR of Nivolumab combined with chemotherapy in the ITT population was 24%, while the pCR of the patients treated with chemotherapy alone was only 2.2%, an increase of about 10 fold (overall response = 13.94, 99% confidence interval: 3.49–55.75 (*P* < .0001). Moreover, regardless of the stage of the disease, the pCR rate and the depth of pathological remission improved significantly. In terms of secondary end points, the MPR of patients who had received Nivolumab combined with chemotherapy before surgery was 4 times higher than that of patients treated with chemotherapy alone (36.9% vs 8.9%; overall response = 5.70, 99% confidence interval: 3.16–10.26). Exploratory analysis showed that the ctDNA clearance rate in the nivolumab combined with chemotherapy group exceeded that of the chemotherapy group (56% vs 34%). At the same time, the higher ctDNA clearance rate also suggested a higher pCR rate. This indicated that there was a strong correlation between the pCR clearance rate and pCR. Combined with the clear predictive effect of pCR and ctDNA clearance on long-term survival in the NADIM study, the pCR benefits of neoadjuvant therapy with immunotherapy plus chemotherapy compared with chemotherapy alone are to be expected, as is the higher proportion of ctDNA eventually translating into long-term survival benefits.

### 4.2 Related adverse reactions from nivolumab combined with chemotherapy in neoadjuvant therapy for NSCLC

Although nivolumab has produced numerous positive results in clinical trials, we should also pay attention to the adverse effects of immunotherapy, which can affect multiple organs in the body, such as the liver, lungs, skin, eyes, endocrine system and gastrointestinal tract.^[19,20]^ The main adverse reactions of nivolumab combined with chemotherapy in neoadjuvant therapy for non-small cell lung cancer are as follows.

In terms of safety in the Checkmate159 study, treatment-related adverse reactions happened in only 23% of the cases, and only one case was more than grade 3. The study revealed that after immunotherapy, the use of PD-1 blockers before surgery not only improves the response of anti-tumor T cells, but also stimulates the peripheral blood's production of CD8+T cells with memory characteristics, and continuously terminates cancer cells through the T cells’ anti-tumor activity. This reduces the tumor cells’ viability in micrometastases, potentially reducing the likelihood of tumor micrometastasis after surgery, and mitigating the risk of recurrence. In the NADIM study, the incidence of adverse reactions of degree 3 or above was 30%. No adverse reactions resulted in discontinuation of treatment, delayed surgery or death during the neoadjuvant therapy stage. During the postoperative adjuvant therapy stage, 14% of the patients terminated the treatment due to adverse reactions. In the CheckMate816 study, the patients tolerated the neoadjuvant immunotherapy, and there was no significant increase in surgical complications. The proportions of surgery-related adverse event reported in the nivolumab combined with chemotherapy group and the chemotherapy group were 41% and 47%, respectively, and the surgery-related adverse event for grades 3 to 4 were 11% and 15%, respectively.

Several existing studies have shown that the safety of neoadjuvant immunotherapy is similar to that of previous neoadjuvant chemotherapy and radiotherapy. Most of the treatment-related adverse events during the nivolumab combined with chemotherapy neoadjuvant therapy were grade 1–2, and there was no death caused by the adverse events. Neoadjuvant nivolumab therapy combined with chemotherapy maintains tolerable safety features and does not hinder surgery feasibility. In exploratory subgroup analysis, the nivolumab plus chemotherapy group's ctDNA clearance rate was higher than that of the single drug chemotherapy group, indicating that it may have been related to pCR. Data from several clinical studies have shown that immunoadjuvant therapy does not affect surgery timing. We expect this to change the traditional lung cancer treatment mode.

## 5 Summary

Lung cancer is the leading cause of cancer death throughout the world, and cases are on the rise due to a variety of factors. A growing number of clinical trials have demonstrated that immunotherapy is essential to lung cancer treatment. The patient in our study was initially diagnosed as being at the locally advanced T4N2M0IIIB stage, with a TPS of only 2%. Before the era of immunotherapy, previous evidence for patients with stage NSCLC showed that without considering radical surgery, the highest level of evidence for NSCLC patients with IIIB was simultaneous radiotherapy and chemotherapy, but the effects have not been satisfactory, especially for patients in China. In this case, combined with the wishes of the patient and the latest research results, we confirmed pCR by radical surgery after 3 rounds of neoadjuvant therapy consisting of nivolumab combined with chemotherapy. This has revealed a potential cure for more patients with lung cancer. Of course, determining whether patients with locally advanced NSCLC with low TPS expression can achieve success in neoadjuvant therapy necessitates further exploration of possible mechanisms and more accurate biomarkers. Whether each of these patients needs 3 rounds of neoadjuvant therapy as immunotherapy combined with chemotherapy is also worthy of further discussion. For example, can patients who receive 2 cycles of neoadjuvant therapy also receive 4 rounds of neoadjuvant therapy? If patients have poor response to 2 cycles of neoadjuvant therapy, should we be more flexible to adjusting the original neoadjuvant therapy regimen? Should we consider changing multiple disciplinary team treatment strategies? These questions could be addressed with more evidence from randomized controlled trial studies.

Although the neoadjuvant therapy model of immunotherapy plus chemotherapy has produced a qualitative leap in the proportion of pCR in non-advanced NSCLC patients, we still need to continue exploring the following issues: Does pCR show long-term benefits in prospective studies on larger populations? Long-term survival is the most important outcome, and we look forward to the CM816 long-term follow-up); Is it necessary for pCR/MPR patients to adjust their surgical plans and methods? For a more accurate population distinction, which population is most suitable for quantifying postoperative assistance with individualized regimens (i.e., biomarkers selected)? Are there specific patients who are exempt from surgery? Is overtreatment a concern? Is the same scheme is used in different types, different stages and different PD-L1 expressions? For each patient, we need to combine the evidence from randomized controlled trial research with patients’ wishes to arrive at a comprehensive diagnosis and treatment strategy so that the patient can be an active participant in their treatment. Only in this way can patients maximize benefits. Based on this case report and related studies, we have reason to believe that the future will bring greater progress in immunotherapy, such that more lung cancer patients will be cured (Supplementary Digital content).

## Acknowledgments

We would like to thank the Department of Radiology, Pathology, and Pharmacy at the Zhongshan Hospital of Traditional Chinese Medicine.

## Author contributions

**Data curation:** Ting Chen.

**Investigation:** Xianhui Zhao.

**Methodology:** Jiaming Wu.

**Resources:** Ao Zhang, Huisi Hong, Lexia Wu, Sihong Lin.

**Resources:** Huatang Zhang.

**Supervision:** Cantu Fang.

**Validation:** Kexin Wang.

**Writing – original draft:** Luzhen Li.

**Writing – review & editing:** Huiqin Lai.

## Supplementary Material

Supplemental Digital Content
