# Supplementary material for: Case report: pathological complete response in an IIIB non-small cell lung cancer patient after preoperative neoadjuvant nivolumab therapy combined with chemotherapy
Source: Medicine (Baltimore). 2022 Jun 10;101(23):e29336. doi: 10.1097/MD.0000000000029336 (PMC9276206; doi:10.1097/MD.0000000000029336)

Figures information

1、Data of initial diagnosis before neoadjuvant therapy

4-22-2021 (Figure 1A): At first diagnosis, the CT showed that the lumpy soft tissue density shadow was in the dorsal segment of the lower lobe of the right lung. The area was about 69 mm × 56 mm, and there were many enlarged lymph nodes in the mediastinum, the length and diameter of which were about 19 mm.


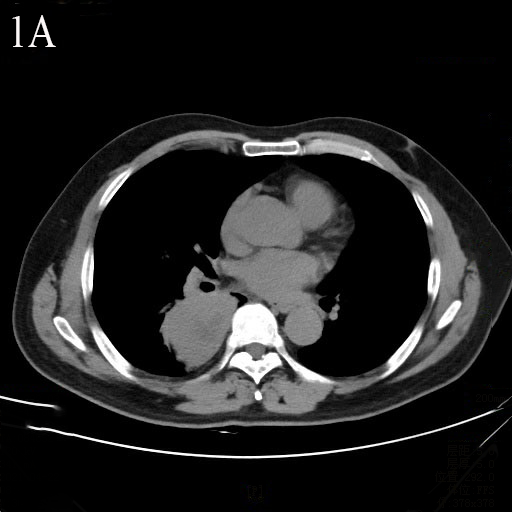

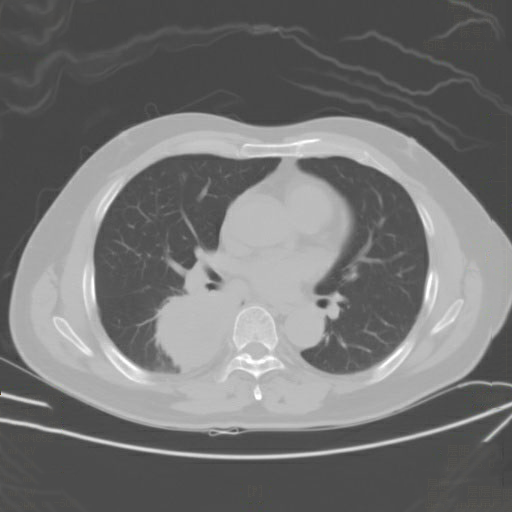


05-01-2021 (Figure 1B (IHC)): Immunohistochemistry: CK (+), Ki67 (60% +), p63 individual cells (+), TTF-1 (-), Syn (-), CD56 (-), CgA (-)

(Figures 1B(PD-L1):): PDL-1 TPS:2%

（On the left is IHC，right is PD-L1）


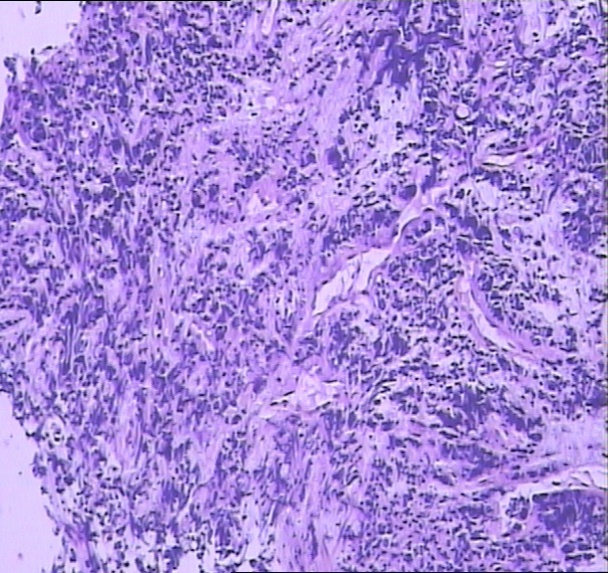

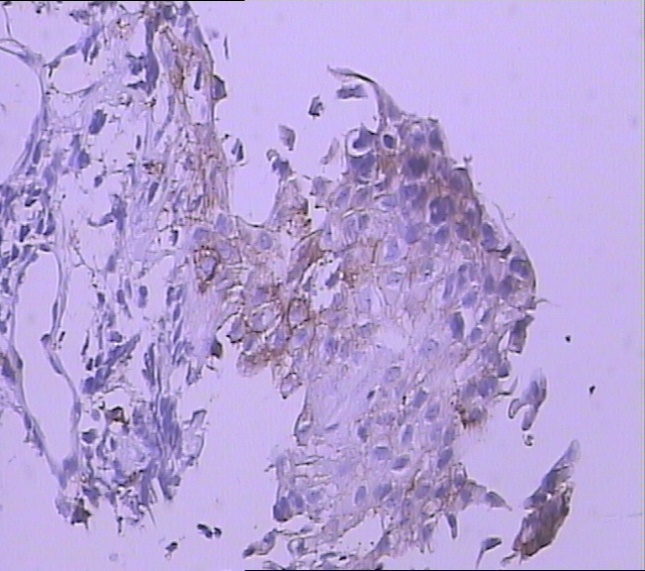


2、Data after neoadjuvant therapy

06-24-2021 (Figure 2A): after 2 courses of neoadjuvant therapy, compared with the chest CT film of 2021-4-22: the tumor in the dorsal segment of the lower lobe of the right lung was significantly smaller than before, the size was about 23mm × 29mm, the edge was smooth, and the surrounding exudate was absorbed. The nodule in the middle lobe of the right lung is the same as before. The mediastinal and right hilar lymph nodes were smaller than before.


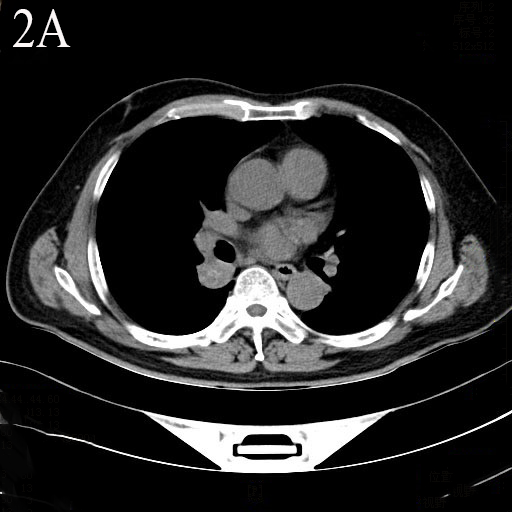

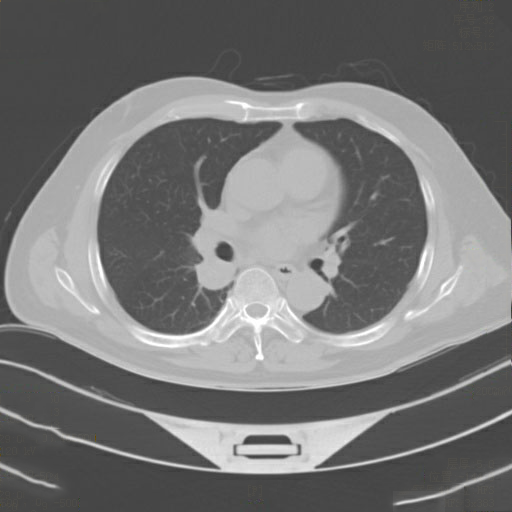


07-03-2021（Figures 2B）:Before the third course of neoadjuvant therapy: the mass continued to shrink，the size is about 18mm × 26mm


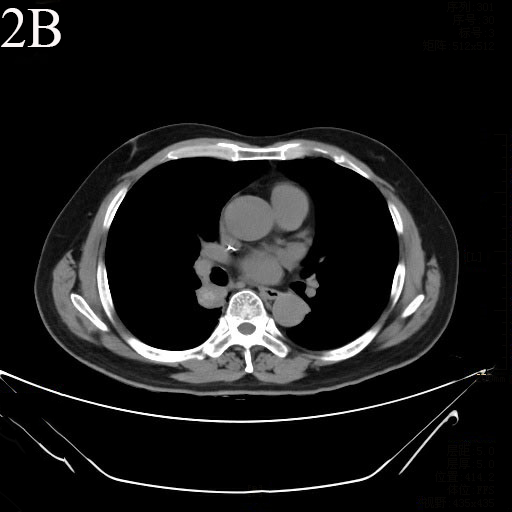

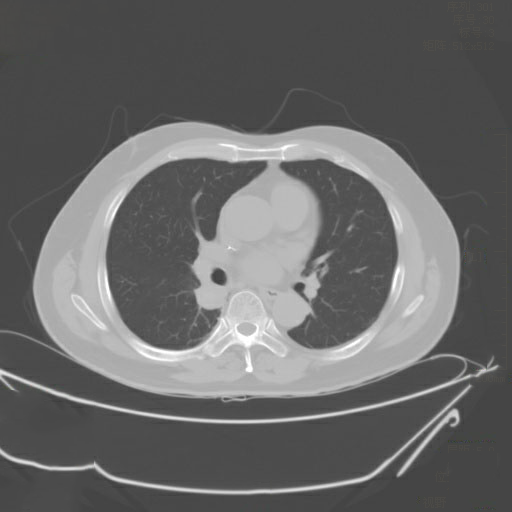


02-18-2022 (Figure 2C): We followed up on the patient every three months, for a total follow-up period of 8 months. The latest images showed no recurrence


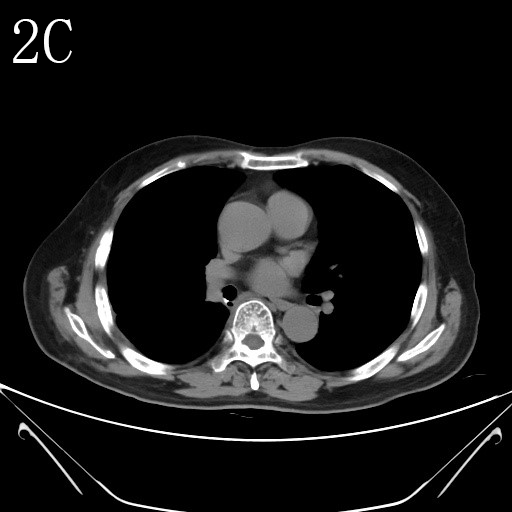

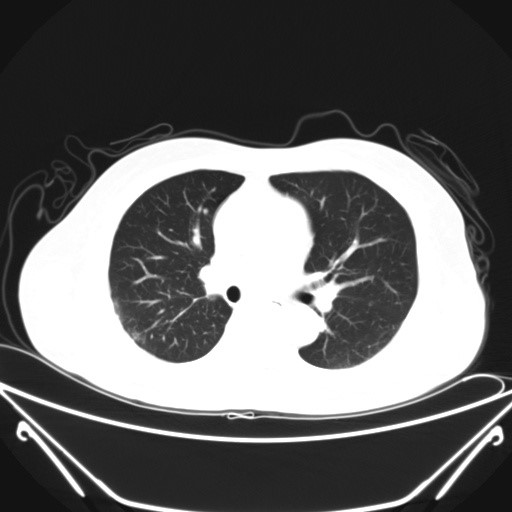


Others:

genetic data


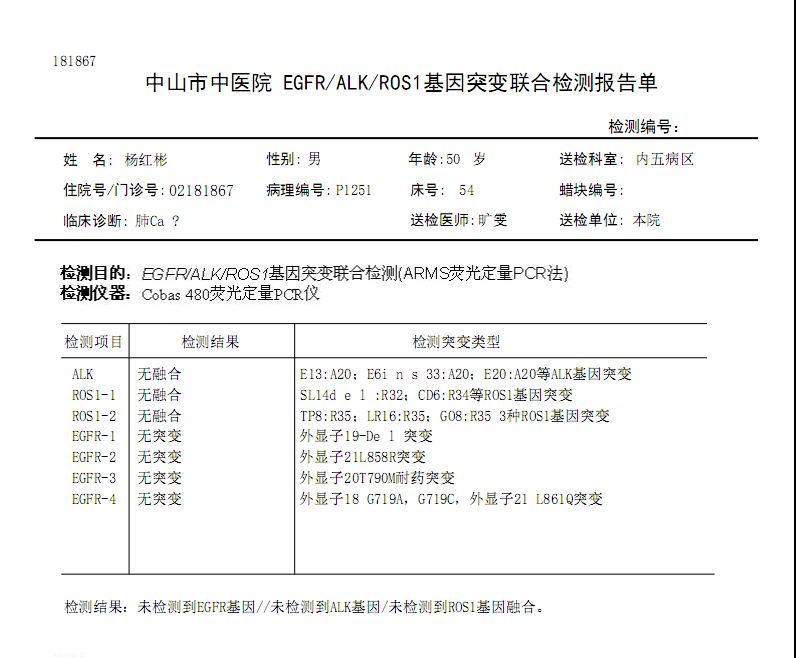


Postoperative pathological data


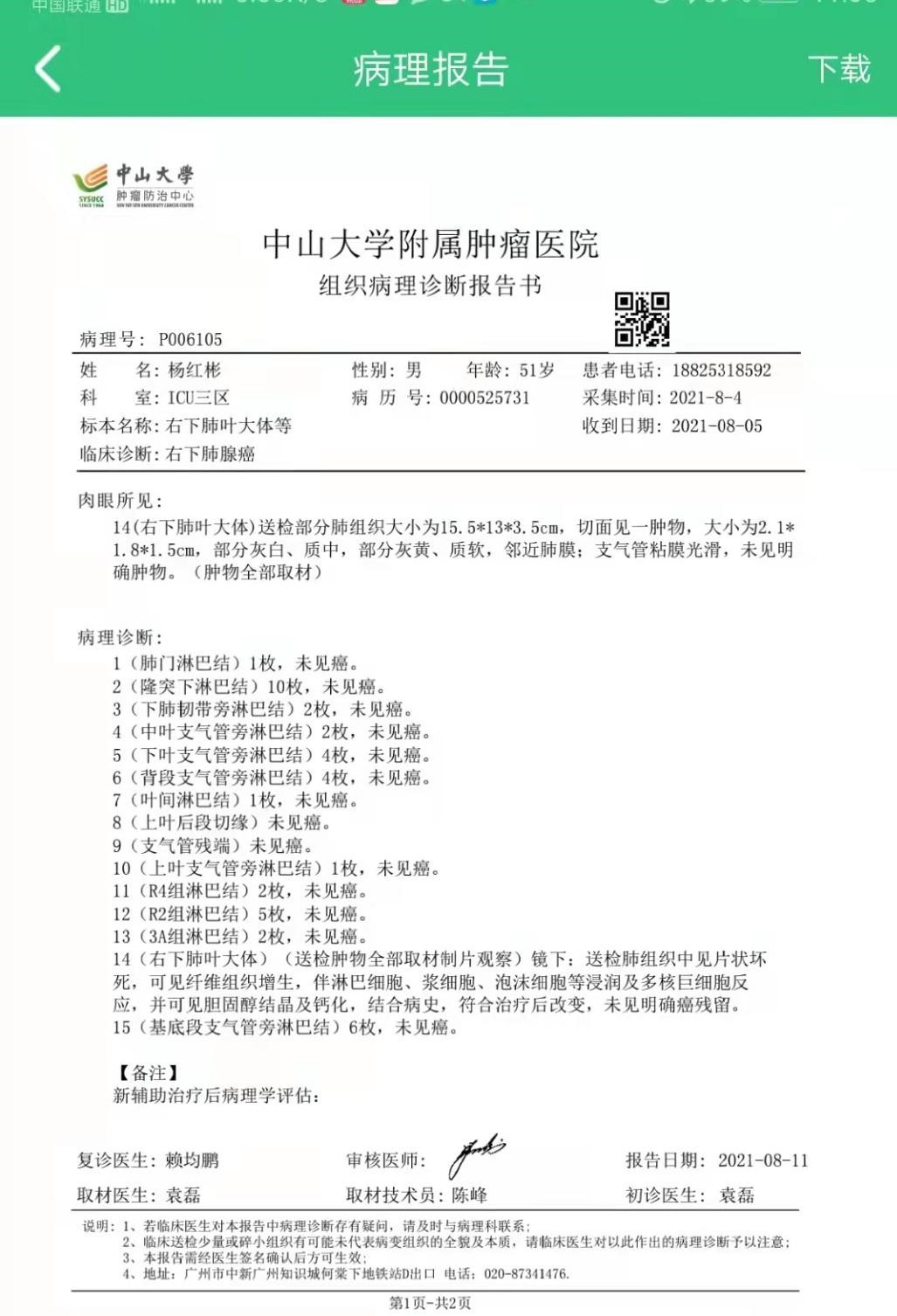

Supplement: Supplemental Digital Content [file medi-101-e29336-s001.docx]
